# CpG Immunotherapy in Chenopodium album sensitized mice: The comparison of IFN-gamma, IL-10 and IgE responses in intranasal and subcutaneous administrations

**DOI:** 10.1186/1476-7961-6-10

**Published:** 2008-09-17

**Authors:** Tahereh Mousavi, Nader Tajik, Maziar Moradi, Masoomeh Fallah Radjabzadeh

**Affiliations:** 1 Department of Immunology, Iran University of medical sciences, Shahid Hemmat highway 14496, Tehran, Iran; 2 Department of Social Medicine, Iran university of Medical Sciences, Shahid Hemmat highway 14496, Tehran, Iran

## Abstract

**Background:**

Mucosal-based immunotherapy has been already used as an alternative form of allergen delivery. In asthma, the poor success rate of immune modulation could be a consequence of inadequate immune modulation in the airways. Previously, we have found that subcutaneous (S.C) co-administration of a homemade allergenic extract from Chenopodium album (Ch.a) pollen and Guanine-Cytosine containing deoxynucleotides (CpG-ODNs) is effective to prevent the inflammatory responses in mouse. In this study we used CpG/Ch.a for immunotherapy of Ch.a-induced asthma and compared the intranasal (I.N) and S.C routes of administration concerning IFN-γ, IL-10 and total IgE responses.

**Methods:**

Ch.a sensitized mice were treated intranasaly or subcutaneously using CpG and Ch.a. extract. IFN-γ, IL-10 and total IgE were measured in supernatant culture of splenocytes and bronchoalveolor lavage (BAL) fluids by ELISA. Student's t test was used in the analysis of the results obtained from the test and control mice.

**Results:**

We found that I.N administration of CpG/Ch.a in sensitized mice significantly increased the production of systemic and mucosal IFN-γ and IL-10 compared to phosphate buffered saline (PBS), Ch.a alone and control ODNs treated sensitized mice (P ≤ 0.001). On the other hand, S.C. route induced the systemic and mucosal IFN-γ in the lower levels than in I.N one, and failed to increase systemic IL-10 induction (P = 0.06). Total serum IgE in CpG/Ch.a treated mice in both routes showed significant decreases compared to three control groups (P ≤ 0.01). The amounts of IgE in BAL fluids were not measurable in all groups.

**Conclusion:**

According to the results of this experiment we concluded that immunotherapy via the I.N co-administration of CpG/Ch.a in comparison with S.C route is more effective to stimulate the mucosal and regulatory responses in Ch.a induced asthma.

## Background

Immunomodulatory agents and their applications in allergic diseases have become one of the most investigating subjects in recent years [1,2]. Because of their potentials in immune response deviations, CpG-ODNs are used to shift immune response toward Th_1 _and regulatory cytokine induction. These cytokines can ultimately lead to prevention or reduction of pathologic features in asthma and other allergic conditions [3,4]. There have been many reports on beneficial properties of CpG motifs used in combination with different antigens from all over the world [5-8]. Furthermore, the route of administration is an attractive subject among these studies [9,10].

In the present study we aimed to compare a number of the immunomodulatory effects of I.N and S.C co-administration of CpG motifs in combination with allergen of Chenopodium album (Ch.a) in Ch.a sensitized mice. The antigen used in this study was a crude allergenic extract prepared from Ch.a pollen which is one of the most common allergenic agents in Iran. Previously we had demonstrated the potentials of this extract to develop an experimentally induced asthma in mouse. We also showed the preventive effects of CpG motifs administered with Ch.a extract at the sensitization stage [11,12].

Since the usage of adjuvant like CpG motifs for mucosal immunotherapy was successful [13], we aimed to compare the potentials of I.N. and S.C administrations of CpG/allergen in mouse model of asthma. In order to compare the effects of different administration route of CpG, we selected a number of immunological parameters such as IFN-γ, IL-10 and IgE. Indeed, the measurement of immune responses regarding T_H_1 and regulatory activities could be the indicators for evaluating and choosing the appropriate route for CpG/allergen co-administration.

## Methods

### Antigen

allergen extract was prepared according to previously reported procedure [14]. Briefly, the pollen grains collected from the flowering Ch.a. plant were immediately vacuum dried at 35°C, purified up to 98% through sieving, defatted with acetone, dried and extracted in PBS (0.02 M, pH = 7.4) overnight at 4°C. The filtered solution then was dialyzed against PBS and sterilized by 0.22 μm filtration.

### Oligonucleotides

The CpG-ODNs contain two CpG motifs (ODN 1826) and control ODNs lacking CpG motifs (ODN, 1826 Control) were purchased from In vivogen, USA. The complete sequences for CPG-ODNs and ODNs control are as follows respectively: 5'-tcc atg acg ttc ctg acg tt-3' and 5'-tcc atg agc ttc ctg agc tt-3'

### Animal immunization

Inbred female BALB/c mice aged in 4–6 weeks were purchased from Razi institute in Iran. All experiments complied with the requirements of the animal care committee of Iran University of medical sciences. Using previously reported protocols [[11,15] and [16]], Mice were immunized on day 1 and 7 IP with 50 μg Ch.a precipitated in 4 mg aluminium hydroxide in 200 μl PBS and followed by aerosol challenge of 1% Ch.a (1 mg Ch.a extract in 100 ml PBS) on days 14 and 16 for 30 min.

### Immunotherapy

For treatment, sensitized mice were divided into I.N and S.C groups. Each group was then randomly subdivided into four (10 in each) as following:

CpG/Ch.a = sensitized mice treated with Ch.a and CpG mixture (50 μg/10 μg respectively). Ch.a = sensitized mice treated with Ch.a alone (50 μg). PBS = sensitized mice treated with PBS. ODN/Ch.a = sensitized mice treated with non CpG ODN control (50 μg/10 μg respectively). I.N treatment was done on day 19, 26 and 33 in I.N group as mentioned above. S.C treatment was done on day 19 and 26 for mice which were considered as S.C group. Finally, all groups of mice were secondly exposed to aerosol allergen (1%) on day 40 and 47 for 30 min.

### Sample preparation

Blood samples were collected from all mice two days after the final antigen challenge on day 49 and separated sera were stored at -20°C for IgE assays. Spleens were also excised on day 49 and single cell suspensions were cultured in complete medium (5 × 10^6 ^cells/ml in RPMI, 10% FCS, 100 U/ml pen/strep) in the presence of 50 μg/ml of allergen for 72 hrs at 37°C in 5% Co2. Cell culture supernatants prepared from all mice were stored at – 80°C for cytokine assays.

Bronchoalveolar lavage fluids were prepared according to previously reported methods [11,12]. Briefly, therachea was canulated and BAL fluids were obtained by lavaging lungs with two 0.5 ml of cold PBS.

### Cytokine assay

IL-10 and IFN-γ were measured in splenocytes culture media and BAL Fluids using mouse IL-10 ELISA set, cat. no. 555252 and mouse IFN-γ ELISA set, cat. no. 5518660 respectively (BD Biosciences, USA). Tests were done according to the manufacturer's recommendations.

IgE assay-ELISA was performed for total serum IgE assay using Becton Dickinson opteia mouse IgE set, cat no.5552448, (BD, USA). According to the manufacturer the lower detection limits of the assay system was 2 ng/ml.

### Statistics

Data were expressed as mean ± SD. Each experiment was repeated twice. Student's t-test was performed for statistic analysis.

## Results

**Figure 1:**
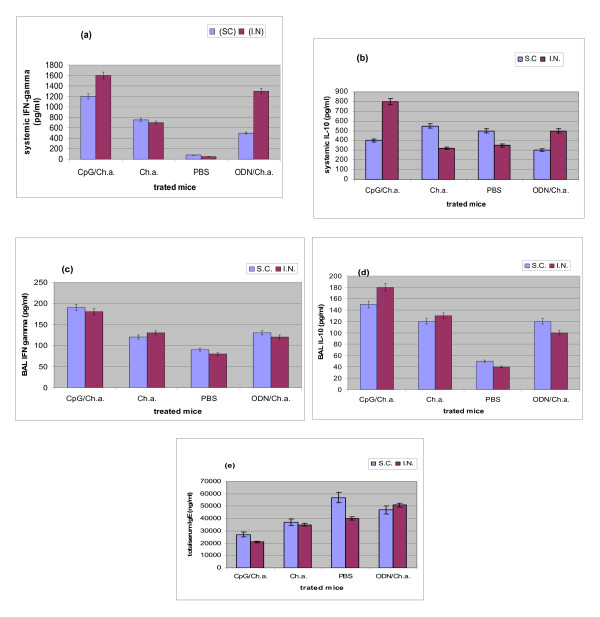
**The comparison of cytokines and antibody levels**. The mean values of systemic and local concentrations of cytokines and IgE antibody measured by ELISA in S.C and I.N CpG/Ch.a treated asthmatic mice in comparison with Ch.a, PBS and ODN treated controls. (a): IFN-γ produced by splenocytes in CpG/Ch.a treated mice are increased in both routes of administrations, (b): IL-10 induced by splenocytes suppressed in S.C and enhanced in I.N routes, (c and d): IFN-γ and IL-10 in BAL fluids are equally increased in both S.C and I.N treated mice with CpG/Ch.a. (e): total serum IgE decreased in mice treated with CpG/Ch.a through I.N or S.C routes. P values in all analysis are as ≤ 0.01.

Our results demonstrated elevated levels of IFN-γ production from splenocytes after both I.N and S.C treatment with CpG/Ch.a compared to Ch.a alone, PBS and CpG/ODN controls (P ≤ 0.001). Furthermore, as presented in Figure 1(a) IFN-γ production from splenocytes in I.N treated mice both in CpG/Ch.a and in ODN/Ch.a treated mice are significantly higher than those in Ch.a alone and PBS treated mice.

Respecting IL-10, I.N treatment with CpG/Ch.a showed significant increases in systemic IL-10 levels compared to all controls (P ≤ 0.001). But, the mean systemic concentrations of this cytokine in S.C treated mice with CpG/Ch.a were lower in comparison with those in Ch.a and PBS controls, and higher than those in ODN/Ch.a controls (P ≤ 0.01) as shown in figure 1(b).

In order to analyze the effect of I.N versus S.C administration of CpG in mucosal responses we measured the IL-10 and IFN-γ in BAL fluids of different groups of mice. We found that IFN-γ and IL-10 were both increased in BAL fluids and results demonstrated that independent to administration route, CpG/Ch.a treatment significantly increased the production of these cytokines (P ≤ 0.001). As shown in figure 1(c) and 1(d), the route of administration did not affect the production of cytokines in BAL fluids and no significant changes were shown in cytokine levels between either route of administration (P = 0.06).

Total serum IgE in CpG/Ch.a treated mice decreased significantly compared to control groups independent to administration routes (P ≤ 0.01). But the amount of IgE was not detectable in BAL fluids as shown in figure 1(e).

## Discussion

At the present time there are many reports on CpG-ODNs used with different allergens [17,18]. But, there is no report in the literature regarding the mechanisms for immunomodulatory effects of CpG-ODNs on Ch.a induced asthma. In this study we compared the effectiveness of CpG components for I.N and S.C immunotherapy of mice sensitized by Ch.a, allergenic extract. This extract was made from one of the common allergenic pollen in our country. For this goal we evaluated a number of systemic and local immunomodulatory effects of CpG motifs in Ch.a induced asthma. We measured IFN-γ, IL-10 and IgE as the Th1, T-reg and Th2 like responses, respectively. In consistence with other reports [6,10], we found in our study the increased IFN-γ production in splenocytes culture supernatants as well as in BAL fluids after CpG/Ch.a therapy. These results indicated the potentials of CpG motifs to enhance the systemic and local Th_1 _like responses in Ch.a sensitized mice. However, concerning IFN-γ production, our results indicated that I.N administration of CPG motifs was more effective than S.C route. Thus, according to a number of studies [19,20], we can suggest that this effect could be attributed to expression of TLR-9 and also the presence of dendritic cells in the nasal epithelium.

Recently, induction of IL-10 has been proposed as an important mechanism of immunotherapy [15,21]. Similarly, our data showed that I.N treatment of mice increased the systemic and mucosal levels of IL-10 as a regulatory cytokine. However, our results indicated that S.C administration of CpG/Ch.a enhanced the local elevation of this cytokine but failed to increase the systemic IL-10. Interestingly, we found not only the elevation, but also the reduction in systemic IL-10 in S.C treated mice. This effect indicates the potentials of I.N but not S.C route to stimulate both the spleen and lung lymphocytes to produce IL-10 cytokine. Therefore, based on the study of Macubas et. al [13] which reported that respiratory tolerance is mediated by IL-10 producing dendtitic cells in lung leading to development of T-reg cells, we can suggest that I.N administration of CpG/Ch.a may activate the T-reg populations in lung and spleen of Ch.a sensitized mice. This result could indicate the advantage of I.N route of administration in CpG/Ch.a therapy of asthmatic mice. On the other hand, we found the significant increases in IL-10 levels after I.N therapy not only with CPG, but also with non-CPG containing ODNs. These effects for CpG negative control were not observed in S.C. administration route. According to Sano et al [22] who observed that non-CpG ODNs trigger Th2-biased immune stimulation, it seems that immunoregulatory effects of DNA components could be partly due to development of T-reg responses, especially when they are used through mucosal surfaces. Considering the reports about the participation of regulatory cells and molecules in the down modulation of immune responses in asthma [21], we suggest that intranasaly co-administration of allergenic extract from Ch.a pollen and CpG motifs in Ch.a induced asthma activates the systemic and local IL-10 producing T-reg cells. However, further studies are necessary to show that the T-reg cells in the lung are responsible for the induction of tolerance through the I.N administration of CpG/Ch.a. in sensitized mice.

Regarding T_H_2 responses, our study indicated a decrease in total serum IgE following the CpG immunotherapy. In contrast to Mo JH [17] we and Suzuki et al [23] detected the significant declines in IgE antibodies after CpG therapy. As the IgE detection in BAL fluids was impossible, we would suggest the further evaluation of local Th2 responses such as eosinophilia in the BAL or airway hyper responsiveness.

On the other hand, In the case of route of administration and chemical component of allergen in CPG-based immunotherapy, considering other reports studding on other allergic conditions than on asthma [24], and also according to Suzuki et al [23] reporting the usefulness of CpG-ODNs in intranasal administration for control of allergic rhinitis to Japanese cedar, we showed that for immunotherapy of Ch.a sensitizes mice, nasal route which is the natural way to induce asthma could be more effective than S.C one. Moreover, in consistence with other reports indicating the advantage of allergenic proteins over peptide epitops in immunotherapy [25], our study indicated that the crude allergenic protein which is simply prepared from Ch.a pollens is a suitable material for in vivo application in mice.

## Conclusion

Taken together, treatment of Ch.a induced asthma in mice via the I.N co-administration of CpG motifs with a crude extract of Ch.a pollen would be very effective compared to S.C route of administration. However, the further studies are needed to indicate the beneficial effects of this protocol in the field of human immunotherapy.

## Competing interests

The authors declare that they have no competing interests.

## Authors' contributions

TM and NT have designed the study and performed experiments, MM performed the statistical, MFR have worked on the draft versions of the paper. All authors have revised the final version.
